# Potential Roles of Extracellular Vesicles in Murine Tear Fluids in the Physiology of Corneal Epithelial Cells In Vitro

**DOI:** 10.3390/ijms26157559

**Published:** 2025-08-05

**Authors:** Saya Oya, Kazunari Higa, Tomohiro Yasutake, Risa Yamazaki-Hokama, Masatoshi Hirayama

**Affiliations:** 1 Department of Ophthalmology, Faculty of Medicine, Fukuoka University, Fukuoka 814-0180, Japan; 2 Department of Ophthalmology, Tokyo Dental College Ichikawa General Hospital, Chiba 272-0824, Japan; 3 Department of Ophthalmology, School of Medicine, Keio University, Tokyo 160-8582, Japan

**Keywords:** tear fluid, exosome, extracellular vesicles, dry eye disease, corneal epithelium

## Abstract

Biological extracellular vesicles in tear fluids, such as exosomes, are thought to have physiological functions in the management of healthy ocular surface epithelium, including corneal epithelium. However, the physiological roles of tear extracellular vesicles in the ocular surface remain unclear. In this study, we investigated the physiological function of tear extracellular vesicles in mouse tear fluids in the ocular surface epithelium in vitro. Morphological analysis of the isolated extracellular vesicles from mouse tear fluids was performed using nanoparticle tracking analysis and transmission electron microscopy. The identified particles were characterised by immunoblotting for exosomal markers. After confirming the uptake of tear exosomes in cultured corneal epithelial cells, gene expression changes in mouse cultured corneal epithelial cells after tear exosome treatment were analysed. Immunostaining analysis was performed to confirm cell proliferation in the cultured corneal epithelial cells with tear exosome treatment. Tear fluids from mice contain nanoparticles with exosome-like morphologies, which express the representative exosomal markers CD9 and TSG101. The extracellular vesicles can be taken up by cultivated murine corneal epithelial cells in vitro and induce expression changes in genes related to the cell cycle, cell membranes, microtubules, and signal peptides. Treatment with the tear extracellular vesicles promoted cell proliferation of cultured murine corneal epithelial cells. Our study provides evidence that murine tear fluids contain extracellular vehicles like exosomes and they may contribute to the maintenance of the physiological homeostatic environment of the ocular surface.

## 1. Introduction

Extracellular vesicles (EVs) have been identified as intercellular signalling factors contained in body fluids that contribute to various cell physiological and pathological processes, including tumour metastasis and neurological diseases [1,2,3,4]. EVs represented by exosomes are cell-derived vesicles composed of a lipid bilayer membrane and contain various biological substances, such as nucleic acids [e.g., mRNAs and microRNAs (miRNAs)] [1,5]. EVs are characterised by the expression of various exosomal markers, including membrane proteins, such as CD9, CD63, and multivesicular body synthesis-related proteins, such as TSG101 and Alix [6]. Exosomes are responsible for specific cell–cell communication through uptake into target cells and are involved in the regulation of biological functions of organs and disease-related processes, such as neovascularisation for cancer metastasis [3]. Current exosome research has been diverse and widely used for medical applications.

Researchers have recently focused on the role of EVs in ocular diseases, including glaucoma and retinal diseases [7,8]. For example, EVs and exosomes in anterior chamber fluids, which are derived from cells, have been shown to be involved in the disease mechanism of glaucoma by modulating signalling pathways in trabecular meshwork cells [7]. In ocular surface diseases, studies on corneal damage have suggested the involvement of corneal mesenchymal cell-derived exosomes in the pathological processes of corneal keratitis, such as wound healing and myofibroblastic transformation [9]. EVs and exosomes have been recognised as critical factors that affect the pathological processes of ocular diseases [10]. Elucidation of novel functions of exosomes will lead to a better understanding of the relationship between exosomes and eye diseases and help develop novel clinical treatments and diagnostic methods.

Tear fluids on the ocular surface maintain the homeostatic microenvironment of the ocular surface epithelium, including transparency and barrier function [11]. A sufficient volume of tear fluid must be retained on the ocular surface because the stable tear film forms a smooth optical surface. At the same time, tear fluids are also thought to contain extracellular vesicles, including exosomes that are needed for physiological corneal epithelial cells. However, the existence of exosomes in mouse tear fluids and their physiological roles on the ocular surface are not fully understood.

In this study, we investigated the physiological function of tear exosomes in the ocular surface epithelium in vitro by using mouse tear fluids. We successfully identified EVs isolated from tear fluids and characterised them as tear exosomes by immunoblotting for exosomal markers. After confirming the uptake of tear exosomes in cultured corneal epithelial cells, we revealed a wide range of gene expression changes in mouse cultured corneal epithelial cells after tear exosome treatment. We clarified that the expression of cell proliferation markers increased in the cultured corneal epithelial cells with tear exosome treatment by immunostaining analysis. Our current study thus suggests the importance of exosomes in tear fluids in the cell physiology of the ocular surface epithelium.

## 2. Results

### 2.1. Morphological Details of EVs in Tear Fluids

**Figure 1:**
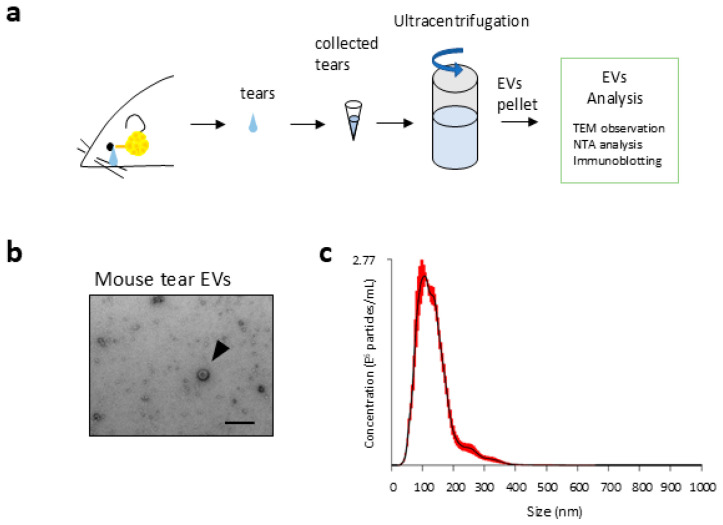
Isolation and morphological analysis of tear extracellular vesicles. (**a**) Schematic representation of the methods used to observe mouse extracellular vesicles from tear fluids. (**b**) Phase-contrast images of the isolated extracellular vesicles derived from mouse tear fluids using electron microscopy (arrowhead) (200 nm, scale bar). (**c**) Size distribution and concentration of extracellular vesicles (EVs) isolated from mouse tear fluid, as determined by nanoparticle tracking analysis (NTA). The *y*-axis indicates particle concentration (particles/mL), and the *x*-axis represents particle diameter (nm). Red error bars indicate ±1 standard error of the mean. The distribution pattern is consistent with small extracellular vesicles, such as exosomes.

The existence of EVs, including exosomes, has been reported in almost all body fluids, such as blood, saliva, and urine; however, the specific physiological functions of exosomes in tear fluids are unknown. Therefore, we first performed morphological analysis of isolated EVs from tear fluids collected from normal mice by an ultracentrifugation method. Electron microscopic observation revealed spherical structures, which is consistent with previously reported morphological characteristics of exosomes from other cells [12] in tear fluids from mice (Figure 1a). According to nanoparticle tracking analysis (NTA), the average concentration of mouse tear exosomes of the final EV preparation was 2.53 ± 0.22 × 10^12^ particles/mL, and we confirmed that the diameter of the particles in mouse tear fluids was 134 ± 7.0 nm (Figure 1b) [12]. These results indicate that tear fluids from mice contain extracellular vehicles with exosome-like morphologies.

### 2.2. Exosomal Marker Expression on EVs in Tear Fluids

**Figure 2:**
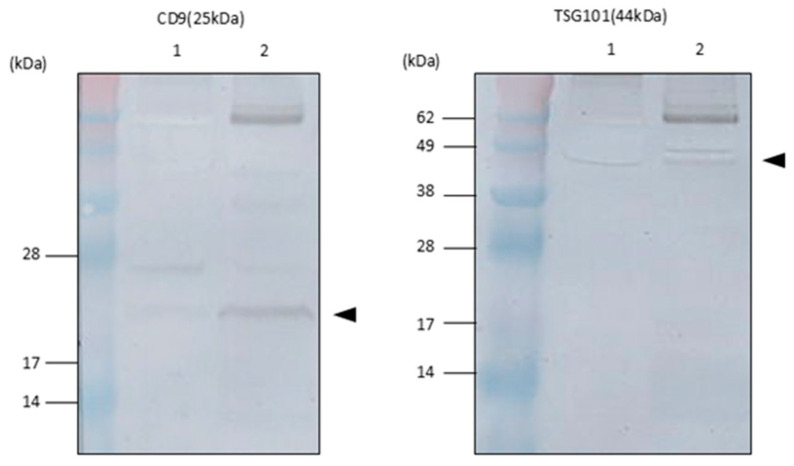
Expression of exosomal markers on EVs in mice tear fluids. (**Left**) CD9 expression in extracellular vesicles from control (lane 1, mouse lacrimal gland extracts as control) and EVs from tear fluids (lane 2, arrowhead). (**Right**) TSG101 expression in extracellular vesicles from control (lane 1, mouse lacrimal gland extracts as control) and EVs from tear fluids (lane 2, arrowhead).

Various proteins, such as tetraspanins, are expressed in the lipid bilayer membrane of exosomes, and their expression has been recognised as representative exosomal markers [12,13]. Therefore, we performed Western blotting analysis of EVs in ultracentrifugation pellets using commercially available antibodies against representative exosomal markers, the tetraspanin molecules CD9 and TSG101 for mouse tear EVs, to confirm the exosomal marker expression in the EVs. Western blotting analysis showed the expression of CD9 and TSG101 in mouse tear EVs (Figure 2). Therefore, we hypothesised that the isolated EVs from mouse tear fluids would contain tear exosomes in this study.

### 2.3. Uptake of Tear Exosomes in Cultivated Corneal Epithelial Cells In Vitro

**Figure 3:**
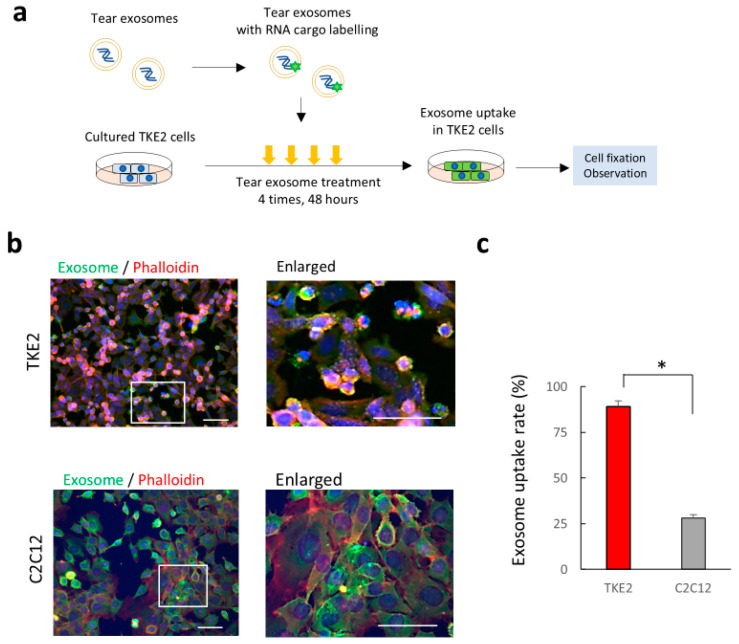
Analysis of uptake in cultured corneal epithelial cells using fluorescein labelled tear exosomes. (**a**) Schematic representation of the methods used to analyse tear exosome uptake in cultured corneal epithelial cells. (**b**) Immunohistochemical analysis of the cultured TKE2 cells (upper panels) and C2C12 cells (lower panels) after a fluorescein-labelled (green) tear exosome treatment. The cells were analysed by immunostaining with specific antibodies for phalloidin (red). Nuclei were stained using DAPI (blue). The boxed area in the left panel is shown at a higher magnification in the right panel. Scale bar, 50 µm. (**c**) The rate of exosome uptake cells in the cultured TKE2 cells and C2C12 cells. The rate of exosome uptake cells in TKE2 cells was significantly higher than in C2C12 cells (* *p* = 0.021, n = 3).

Exosomes in body fluids contribute to cell signalling communication between donor cells and recipient cells through an intake mechanism of exosomes [14]. To prove the uptake of tear exosomes in corneal epithelial cells, we cultured corneal epithelial cells using TKE2 cells [15], a mouse corneal epithelial cell line, with culture media and added tear EVs with fluorescein-labelled RNA. Tracing analysis using fluorescein-labelled tear exosomal RNA showed that exosomal RNA were taken up by TKE2 cells at a rate of 89 ± 3.1% (Figure 3a,b). The uptake rate of tear exosomes in cultured C2C12 cells (mouse C3H muscle myoblasts) was also observed; however, the rate was significantly lower (28 ± 1.9%) than that of corneal epithelial cells at the same transduction condition. These results suggest the possibility of cell signalling communication in corneal epithelial cells through EVs in mouse tear fluids.

### 2.4. Gene Expression Changes in Corneal Epithelial Cells After the Uptake of Tear Exosomes

**Figure 4:**
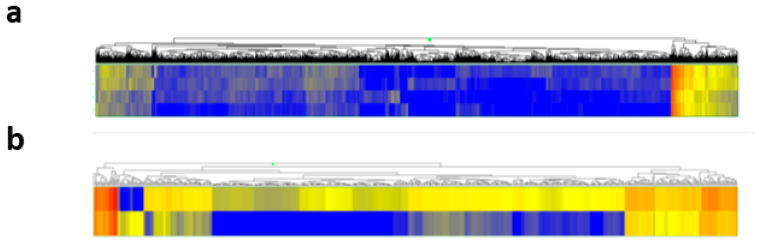
Gene expression changes in cultured corneal epithelial cells with tear exosome treatment. (**a**) Heatmap analysis of all gene expression changes in TKE2 cells with or without tear EV treatment (each kind of sample was duplicated; upper 2 lanes, with the treatment; lower 2 lanes, without the treatment). (**b**) Heatmap analysis of 513 gene expression changes in TKE2 cells, which are increased (fold change ≥ 2.0) after tear EV treatment (upper lane, with treatment, lower lane, without treatment).

Contents in exosomes, including mRNAs and miRNAs, are taken up into recipient cells and function through various biological pathways [16]. We analysed gene expression changes using a microarray of cells to observe biological changes in cultivated corneal epithelial cells with or without tear EV treatment. We first carried out transcriptome analysis to profile mRNA expression changes in TKE2 cells with or without tear EV treatment, using a DNA microarray containing 23,475 oligonucleotide probes corresponding to mouse genes. The comparison of gene expression between cultured TKE2 cells treated or not treated with EVs revealed that 513 genes were significantly upregulated in the EV-treated cells, while 25 genes were significantly downregulated compared to the untreated control (Figure 4a,b). To explore the biological significance of genes upregulated by tear exosome treatment, we performed functional annotation clustering analysis using DAVID. A total of 513 genes with a fold change ≥2.0 were subjected to this analysis. The results identified six annotation clusters (AC1–AC6) with an enrichment score >1.30 and *p*-value < 0.05, which were considered significant. These clusters represent functionally related gene groups associated with the cell cycle (AC1), cell membrane (AC2), transmembrane transport (AC3), cytoskeleton and microtubule organisation (AC4), glycoproteins (AC5), and signal peptides (AC6). These findings suggest that tear exosomes may influence a broad range of cellular processes in corneal epithelial cells. Table 1 shows representative GO terms from the six annotation clusters. The following pathway analysis revealed that two pathways, ectodysplasin A (EDA) signalling and steroid biosynthesis, were significantly enriched (Table 2). These results indicate that tear exosomes induce gene expression changes, which are related to various functions, including the cell cycle and microtubule and transmembrane signalling, in cultured corneal epithelial cells.

### 2.5. Tear Exosomes Induce the Proliferation of Cultivated Corneal Epithelial Cells

**Figure 5:**
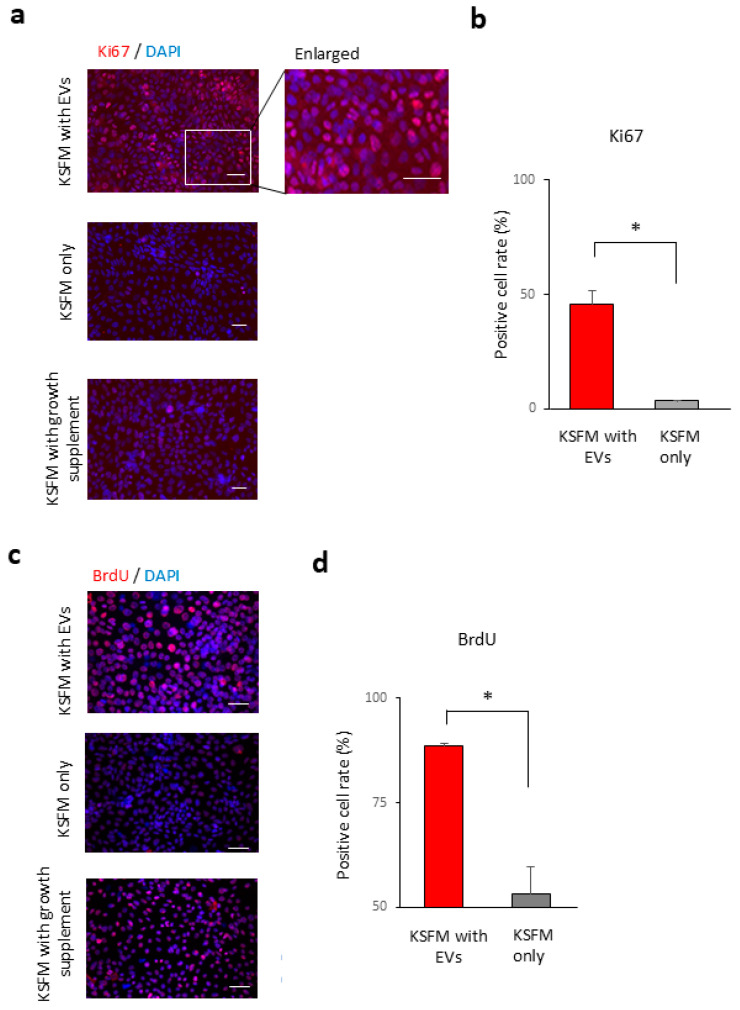
Analysis of cell proliferation in the cultured corneal epithelial cells after tear EV treatment. (**a**) Immunohistochemical analysis of TKE2 cells cultured with basal medium with tear EVs (upper panel and enlarged image), basal medium without tear EVs (middle panel), and medium supplemented with growth factors as a positive control (lower panel). Cells were immunostained with an antibody against Ki67 (red), and nuclei were counterstained with DAPI (blue). Scale bar, 50 µm. (**b**) The rate of Ki67 positive cells in the cultured TKE2 cells. The rate of Ki67 positive cells in TKE2 cells with tear exosome treatment was significantly higher than in TKE2 cells without tear exosome treatment (* *p* = 0.020, n = 3). (**c**) Immunohistochemical analysis of TKE2 cells cultured with basal medium with tear EVs (upper panel), basal medium without tear EVs (middle panel), and medium supplemented with growth factors as a positive control (lower panel). The cells were analysed by immunostaining with specific antibodies for BrdU (red). Nuclei were stained using DAPI (blue). Scale bar, 50 µm. (**d**) The rate of BrdU positive cells in the cultured TKE2 cells. The rate of BrdU positive cells in TKE2 cells with tear EV treatment was significantly higher than in TKE2 cells without the treatment (* *p* = 0.028, n = 3).

The transparency of the cornea is managed by the proliferation of corneal epithelial cells during wound healing processes after corneal epithelial damage [18]. Especially in DED, corneal epithelial damage occurs due to insufficient tear fluids [18]. According to the results of the gene expression analysis described above, we investigated whether tear EVs contribute to the maintenance of ocular surface homeostasis through the regulation of corneal epithelial cell processes, such as cell proliferation. To analyse the roles of tear exosomes in cell proliferation, we performed immunohistochemical (IHC) analysis using antibodies for cell proliferation markers, such as Ki67 and BrdU. Treatment with tear EVs in cultivated TKE2 cells revealed that the rate of Ki67-positive cells in cultured corneal epithelial cells was significantly higher with the treatment (45.4 ± 6.4%) than without the treatment (3.5 ± 0.4%) (Figure 5a,b) (*p* = 0.020). BrdU staining analysis showed that the BrdU-positive cells in cultured corneal epithelial cells were higher after the treatment (88.7 ± 0.5%) than without the treatment (53.3 ± 6.5%) (*p* = 0.028) (Figure 5c,d). These results indicate that tear EVs may contribute to the proliferation of cultured corneal epithelial cells.

## 3. Discussion

In this study, we revealed that tear fluids of mice and humans contain exosomes expressing representative exosomal markers, and intercellular signalling by mouse tear exosomes has a potential role in regulating physiological functions, including cell proliferation, through various gene expression changes in corneal epithelial cells in vitro. These findings indicate a novel function of tear EVs that contributes to the maintenance of the physiological homeostatic environment of the ocular surface.

Tear fluids are indispensable for the ocular surface epithelium to maintain a smooth optical surface for better visual function [19,20]. A shortage of tear fluids on the ocular surface causes DED, which leads to corneal epithelial damage, resulting in a severe decrease in quality of life due to impaired visual function [18,21]. Artificial tear drops have been developed as alternative aqueous tear water to cure DED [22]. Recent findings revealed that exosomes, which are abundant in body fluids, are responsible for cell–cell communication by delivering proteins and nucleic acids, such as RNA, to recipient cells [1,23]. These concepts predict that EVs and exosomes in tear fluids are involved in the maintenance of homeostasis of the corneal epithelium and disease mechanisms of DED. However, the involvement of tear EVs in corneal epithelial cell physiology is unclear [7]. Our study reported that tear EVs, including exosomes, may be functional substances in tear fluids that may help regulate the proliferation of corneal epithelial cells in vitro.

EVs, including exosomes, which are generally isolated by ultracentrifugation from body fluids, are nanoparticles produced by many different cells [3]. Common features of exosomes include a typical morphology, such as vesicles lined by a lipid bilayer, a homogenous size of approximately 100 nm in diameter, and expression of specific proteins, such as tetraspanins, including CD9 and CD63, and others with endocytic origins, such as TSG101 [2]. Exosomes have been reported in almost all biological fluids, such as blood [24], urine [25], saliva [26], breast milk [27], cerebrospinal fluids [28,29], sperm [30], and malignant effusions [31]. However, details on EVs and exosomes in mouse tear fluids are lacking [32]. In this study, we successfully observed nanoparticles in tear fluids from mice, consistent with similar morphologies of exosomes as described previously [12] and confirmed the expression of exosomal markers, suggesting the existence of exosomes in the tear fluids of mice. Further identification of exosomal marker expression in mouse tear fluids requires the development of available antibodies. Investigation on changes of expression profiles of exosomal markers in tear exosomes in diseased status, such as DED, would provide interesting information as a future study [33].

Exosomes contain a wide range of genetic materials, e.g., functionally active RNAs, such as mRNA and miRNA, for intercellular signalling, which are mostly involved in the cell cycle, angiogenesis, differentiation, and DNA histone modification under both physiological and pathological conditions, including tumorigenesis [14,34,35]. In this study, the labelled RNAs in tear exosomes were transferred to cultured corneal epithelial cells at a high rate, suggesting the possibility of a cell–cell communication network in cells with EVs in tear fluids.

Reliable next-generation sequencing technology for nucleic acids in tear exosomes has been difficult due to the volume limits (small amount) of tear fluids, especially in mice. Our in vitro model using cultured mouse corneal epithelial cells with our purified tear EVs treatment could shed a light on the role of tear EVs on ocular surface by using microarray analysis. Our findings clarify the various gene expression changes in cultured corneal epithelial cells due to tear EV treatments. The EDA signalling pathway, which is significantly enriched in cells treated with tear EVs (Table 1), regulates the induction, morphogenesis and maintenance of epidermal structures, such as teeth, hair follicles, sweat glands, and several other glands, through NF-kB-mediated regulation of Wnt stimulation or inhibition and the sonic hedgehog (SHH) and RelB pathways [36]. Our results indicate that tear EVs, including exosomes, promote cell proliferation of corneal epithelial cells through these signalling pathways. Further analysis of the effects of tear EVs and exosomes on a more physiologically mature cell environment using an in vivo model should be performed. Our in vitro experimental model may be appropriate for analysing the fundamental roles of tear EVs and exosomes on the ocular surface epithelium because it excludes the influence of peripheral tissues, including the corneal stroma. These findings suggest that tear EVs may have a variety of effects on the physiology of corneal epithelial cells.

A limitation of our study is the potential contamination from other tear components during the process of isolating EVs or exosomes, which cannot be completely ruled out. Ultracentrifugation is one of the most reliable methods for isolating EVs from small-volume samples, such as tear fluids. However, to achieve more precise isolation in the future, it will be necessary to compare results using alternative methods, such as adsorption-based techniques. While our study demonstrated a difference in exosome uptake between cell types, the mechanistic basis for this observation remains to be elucidated. The significantly higher uptake of tear exosomes by TKE2 cells may suggest a cell-type-specific mechanism influenced by surface characteristics of both EVs and recipient cells [37,38,39]. Future studies employing molecular profiling and endocytic pathway inhibition are needed to clarify the receptors or pathways involved in the differential uptake of tear-derived EVs. Further refinement of isolation protocols and additional marker examination are warranted, particularly for isolating EVs from limited sample volumes. Future studies on functional abilities of EVs in tear fluids in vivo to examine whether tear-derived EVs can exert protective or modulatory effects on the ocular surface under physiological or pathological conditions, such as dry eye or corneal injury, will provide interesting information.

## 4. Materials and Methods

### 4.1. Ethical Statements

This study was approved by the Animal Care and Experiment Committee of the Tokyo Dental College Ichikawa General Hospital (approval number: 207702). C57BL/6 mice were purchased from Charles River Laboratories (Yokohama, Japan). The care and handling of the animals were performed in accordance with the NIH guidelines.

### 4.2. Tear Fluid Collection

From the mice, 5 μL of tear fluid was collected from the eyelid margin without touching the eye using a 0.5 μL micropipette (Drummond Scientific, Broomall, PA, USA), 20 min after intraperitoneal injection of 300  μg of pilocarpine kg^−1^ body weight after washing the eye surface with PBS. The samples were pooled as described previously [40]. Each animal was anaesthetised with an intraperitoneal injection of medetomidine (0.15 mg/kg), midazolam (2 mg/kg), and butorphanol (2.5 mg/kg) before the procedures.

### 4.3. Isolation of EVs

Isolation of EVs from the samples was performed by the differential ultracentrifugation method, as described below [41]. After the addition of 800 µL of PBS, 40 µL of pooled mouse tear samples was centrifuged at 300× *g* for 10 min and 2000× *g* for 10 min. After filtration through a 0.2 µm Millipore filter to remove cell debris, the collected supernatant was centrifuged again at 10,000× *g* for 30 min, and the supernatant was centrifuged in an ultracentrifuge at 100,000× *g* for 70 min to remove proteins and contaminates. After PBS washes, the sample was centrifuged again in an ultracentrifuge at 100,000× *g* for 70 min. All procedures were performed at 4 °C. The pellet containing extracellular vesicles, including exosomes, was used after adding 100 µL of PBS with 1 µL of EV-Save (Fujifilm Wako, Tokyo, Japan) on ice.

### 4.4. Morphological Analysis of Nanoparticles

Exosome samples were diluted to a concentration of 10^8^–10^9^ particles/mL in Milli-Q water for analysis. The size and concentration of the exosomes were determined through nanoparticle tracking analysis using a Nano Sight LM10 system (Malvern Panalytical, Ltd., Malvern, UK). Images for analysis of the Brownian motion were obtained five times in 60 s, and the particle size and particle concentration were calculated. Images of the particles were obtained using transmission electron microscopy (H-7600, Hitachi, Tokyo, Japan) and taken using an AMT XR16S-R CCD camera (Hitachi).

### 4.5. Western Blot Immunoblotting

Isolated EV samples (2.0 × 10^10^ particles/per lane) were separated on a polyacrylamide gel before being transferred to a PVDF membrane. The blotting membrane was blocked with normal goat serum (Vectastain ABC Kit; Vector Laboratories, Burlingame, CA, USA) and incubated with CD63 antibody (Abcam, Cambridge, UK) and with TSG101 (GeneTex, San Antonio, TX, USA) and CD9 (Abcam), followed by incubation with biotinylated secondary antibody (Vector Laboratories). The proteins were detected using a DAB substrate kit (Vector Laboratories).

### 4.6. Cell Culture and Analysis of Exosome Uptake

TKE2 is a murine limbal/corneal epithelium progenitor cell line [15]. TKE2 cells were maintained in defined keratinocyte serum-free medium (KSFM; Gibco-Invitrogen Corp., Carlsbad, CA, USA) supplemented with 1% penicillin/streptomycin and growth supplement supplied by the manufacturer until use. C2C12 cells (ECACC catalogue no. 91031101; mouse C3H muscle myoblasts) were cultured in DMEM with 10% FBS supplemented with 1% penicillin/streptomycin. Cell cultures were incubated at 37 °C under 95% humidity and 5% CO_2_, and the culture medium was changed every 3 to 4 days. For the assessment of exosome uptake by TKE2 cells, 1 × 10^4^ cells of TKE2 cells and C2C12 cells with KSFM media were cultured on a 4-well Nunc Lab-Tek chamber slide system (Thermo Fisher Scientific, Waltham, MA, USA). Mouse tear EVs (2 × 10^12^ particles), which were labelled with SYTO RNASelect Green Fluorescent Cell Stain (Thermo Fisher Scientific) according to the manufacturer’s protocol, were applied to the culture media every 12 h for 2 days. After exosome treatment, the cells were fixed with 4% paraformaldehyde (PFA) (Wako, Osaka, Japan) for 20 min. After the cells were washed with PBS, they were incubated with 0.1% Triton X-100 for 5 min. After the cells were washed again with PBS, they were incubated with Alexa Fluor 594 phalloidin conjugate (Thermo Fisher Scientific) for 20 min for actin staining according to the manufacturer’s protocol. After the samples were stained with 4′,6-diamidino-2-phenylindole (DAPI) (Dojindo Laboratories, Tokyo, Japan), images were obtained using a florescence microscope (Axioplan 2 imaging, Carl Zeiss, Inc., Thornwood, NY, USA). To quantify exosome uptake, images were acquired from three independent experiments (n = 3), and the number of cells showing green fluorescence (SYTO RNASelect-positive) was counted manually under a fluorescence microscope. The total number of cells was determined based on DAPI staining. The uptake ratio was calculated as the percentage of SYTO-positive cells among the total DAPI-positive cells for each image field.

### 4.7. BrdU Labelling

TKE2 cells (10^4^ cells/well) with or without exosome treatment, which were prepared as described above, were incubated with BrdU (final, 10 μM) for 2 h. After fixation with cold acetone at room temperature (RT) for 5 min, the cells were treated with 2 N HCl at RT for 30 min, and BrdU was detected by immunocytochemistry as described below. Ten randomly selected visual fields in each group were photographed, and the rate of BrdU-positive cells was calculated. Three independent experiments were performed.

### 4.8. Immunostaining

For an assessment of cell proliferation after exosome treatment, we prepared TKE2 and C2C12 cells cultured with DKSFM without growth supplement supplied by the manufacturer. Isolated mouse tear exosomes or PBS was applied to the DKSFM without supplementation every 12 h for 2 days. For comparison, we also prepared TKE2 cells cultured with DKSFM with growth supplement supplied by the manufacturer. The cells treated with or without exosomes were analysed by immunostaining for the proliferation marker Ki67. The treated cells were rinsed with PBS and fixed with cold acetone (Wako) for 5 min. After the cells were blocked with 10% normal donkey serum (Chemicon International, Inc., Temecula, CA, USA) at RT for 1 h, they were incubated with Ki67 [MIB-1 (1:50), Dako Cytomation, Glostrup, Denmark] and 5′-bromo-2′-deoxyuridine (BrdU) (BU1/75 (1:50), Abcam) at RT for 1.5 h. After three washes with phosphate-buffered saline for 5 min, the cells were incubated with a rhodamine-conjugated donkey anti-mouse IgG antibody (1:100; Jackson ImmunoResearch Laboratories, West Grove, PA, USA). After three additional washes with PBS, the cells were incubated with 1 µg/mL DAPI (Dojindo Laboratories) at RT for 5 min. Finally, the cells were washed three times in PBS and coverslipped using aqueous mounting medium (Fluoromount/Blue; Diagnostic BioSystems, Pleasanton, CA, USA). Images were acquired using an Axioplan 2 imaging microscope (Zeiss, Oberkochen, Germany). Ten randomly selected visual fields in each group were photographed, and the rate of Ki67-positive cells was calculated. Three independent experiments were performed.

### 4.9. Microarray Data Analysis

To assess transcriptomic changes in corneal epithelial cells following EV uptake, total RNA was extracted from TKE2 cells treated or not treated with tear-derived EVs. The extracted RNA samples were analysed using a Mouse Oligo chip 24 k (1-colour array) and scanned with the 3D-Gene Scanner 3000 (Toray, Tokyo, Japan). This oligonucleotide-based DNA microarray includes 23,475 probes representing mouse genes and is used to profile mRNA expression levels. After normalisation, differentially expressed genes were identified by comparing the EV-treated and untreated samples. Genes with a minimum fold change of ≥2.0 were selected for downstream functional analysis. Functional annotation enrichment analysis of these genes was performed using the DAVID Bioinformatics Resources (https://davidbioinformatics.nih.gov/, accessed on 1 May 2025)). Gene Ontology (GO) terms—encompassing molecular function, biological processes, and cellular components—and pathways with a *p*-value < 0.05 were considered significantly enriched. Annotation clusters with an enrichment score > 1.30 were considered biologically significant and grouped into categories based on DAVID’s classification.

### 4.10. Statistical Analysis

Statistical analysis was performed using BellCurve for Excel (Social Survey Research Information Co., Ltd., Tokyo, Japan). Student’s *t*-test was used for comparative analysis of the rate of exosome uptake and the rate of immunostaining-positive cells. A *p* value < 0.05 was considered statistically significant.

## 5. Conclusions

The current study provides novel evidence for the potential roles of EVs in mouse tear fluids in corneal epithelial cell physiology in vitro. Further studies on the identification of miRNA subtypes in tear exosomes using next-generation sequencing and the involvement of tear EVs and exosomes in normal conditions or diseases, such as DED, inflammatory conditions such as Sjogren’s syndrome, and ageing in humans, should be conducted in the future.

## Figures and Tables

**Table 1:** Representative GO terms by functional annotation cluster analysis of the 513 genes, which were increased (fold change ≥ 2.0) after tear exosome treatment. AC1, AC2, AC3, and AC5 contain GO terms from AC1–AC6, which represent functional annotation clusters generated by DAVID from 513 upregulated genes (fold change ≥ 2.0) following tear exosome treatment [17]. Enrichment score > 1.30 and *p*-value < 0.05 were considered significant.

AC1 (Enrichment score: 2.77)	
GO Term Biological Process	*p*-value
GO:0007049 cell cycle	0.007
GO:0051301 cell division	<0.001
GO:0007067 mitotic nuclear division	<0.001
GO:0007059 chromosome segregation	0.009
GO:0000070 mitotic sister chromatid segregation	<0.001
GO Term Cellular Component	*p*-value
GO:0005694 chromosome	0.041
GO:0000776 kinetochore	<0.001
GO:0000775 chromosome, centromeric region	0.002
GO:0000922 spindle pole	0.001
GO:0000777 condensed chromosome kinetochore	0.001
GO:0000780 condensed nuclear chromosome	0.005
AC2 (Enrichment score: 2.27)	
GO Term Biological Process	*p*-value
GO:0005886 plasma membrane	0.015
AC3 (Enrichment score: 1.86)	
GO Term Cellular Component	*p*-value
GO:0016020 membrane	0.015
GO:0016021 integral component of membrane	0.036
AC5 (Enrichment score: 1.40)	
GO Term Biological Process	*p*-value
GO:0007018 microtubule-based movement	0.073
GO:0007080~mitotic metaphase plate congression	0.031
GO Term Cellular Component	*p*-value
GO:0005819 spindle	0.028
GO:0005871 kinesin complex	0.019
GO:0005876 spindle microtubule	0.044
GO Term Molecular Function	*p*-value
GO:0008017 microtubule binding	0.022
GO:0008574 ATP-dependent microtubule motor activity	0.028

**Table 2:** Top 10 increased signalling pathways enriched in cells that appear in EVs in mice tear fluids.

Pathway	*p*-Value (Comparison)
Mm_EDA_Signalling_in_Hair_Follicle_Development_WP3652_97556	0.007748972
Mm_Steroid_Biosynthesis_WP55_89970	0.027217017
Mm_Glutathione_metabolism_WP164_85644	0.057912603
Mm_Kit_Receptor_Signaling_Pathway_WP407_69079	0.058914933
Mm_Statin_Pathway_WP1_73346	0.06381421
Mm_Metapathway_biotransformation_WP1251_94721	0.07834384
Mm_Notch_Signaling_Pathway_WP29_79679	0.079050094
Mm_Hedgehog_Signaling_Pathway_WP116_69142	0.08262755
Mm_Chemokine_signaling_pathway_WP2292_97515	0.098608024

## Data Availability

The data that support the findings of this study are available from the corresponding author, M.H., upon reasonable request.

## Abbreviations

The following abbreviations are used in this manuscript:

EVs	Extracellular Vesicles
DED	Dry Eye Diseases
EDA	Ectodysplasin A

## References

[1] Valadi H., Ekstrom K., Bossios A., Sjostrand M., Lee J.J., Lotvall J.O. (2007). Exosome-mediated transfer of mRNAs and microRNAs is a novel mechanism of genetic exchange between cells. Nat. Cell Biol..

[2] Pegtel D.M., Cosmopoulos K., Thorley-Lawson D.A., van Eijndhoven M.A., Hopmans E.S., Lindenberg J.L., de Gruijl T.D., Wurdinger T., Middeldorp J.M. (2010). Functional delivery of viral miRNAs via exosomes. Proc. Natl. Acad. Sci. USA.

[3] Hoshino A., Costa-Silva B., Shen T.L., Rodrigues G., Hashimoto A., Tesic Mark M., Molina H., Kohsaka S., Di Giannatale A., Ceder S. (2015). Tumour exosome integrins determine organotropic metastasis. Nature.

[4] Saman S., Kim W., Raya M., Visnick Y., Miro S., Jackson B., McKee A.C., Alvarez V.E., Lee N.C., Hall G.F. (2012). Exosome-associated tau is secreted in tauopathy models and is selectively phosphorylated in cerebrospinal fluid in early Alzheimer disease. J. Biol. Chem..

[5] Pan B.T., Johnstone R.M. (1983). Fate of the transferrin receptor during maturation of sheep reticulocytes in vitro: Selective externalization of the receptor. Cell.

[6] van Niel G., Porto-Carreiro I., Simoes S., Raposo G. (2006). Exosomes: A common pathway for a specialized function. J. Biochem..

[7] Lerner N., Avissar S., Beit-Yannai E. (2017). Extracellular vesicles mediate signaling between the aqueous humor producing and draining cells in the ocular system. PLoS ONE.

[8] Liu J., Jiang F., Jiang Y., Wang Y., Li Z., Shi X., Zhu Y., Wang H., Zhang Z. (2020). Roles of Exosomes in Ocular Diseases. Int. J. Nanomed..

[9] Samaeekia R., Rabiee B., Putra I., Shen X., Park Y.J., Hematti P., Eslani M., Djalilian A.R. (2018). Effect of Human Corneal Mesenchymal Stromal Cell-derived Exosomes on Corneal Epithelial Wound Healing. Investig. Ophthalmol. Vis. Sci..

[10] Klingeborn M., Dismuke W.M., Bowes Rickman C., Stamer W.D. (2017). Roles of exosomes in the normal and diseased eye. Prog. Retin. Eye Res..

[11] Mathers W.D. (2000). Why the eye becomes dry: A cornea and lacrimal gland feedback model. CLAO J..

[12] Doyle L.M., Wang M.Z. (2019). Overview of Extracellular Vesicles, Their Origin, Composition, Purpose, and Methods for Exosome Isolation and Analysis. Cells.

[13] Thery C., Witwer K.W., Aikawa E., Alcaraz M.J., Anderson J.D., Andriantsitohaina R., Antoniou A., Arab T., Archer F., Atkin-Smith G.K. (2018). Minimal information for studies of extracellular vesicles 2018 (MISEV2018): A position statement of the International Society for Extracellular Vesicles and update of the MISEV2014 guidelines. J. Extracell. Vesicles.

[14] Skog J., Wurdinger T., van Rijn S., Meijer D.H., Gainche L., Sena-Esteves M., Curry W.T., Carter B.S., Krichevsky A.M., Breakefield X.O. (2008). Glioblastoma microvesicles transport RNA and proteins that promote tumour growth and provide diagnostic biomarkers. Nat. Cell Biol..

[15] Kawakita T., Shimmura S., Hornia A., Higa K., Tseng S.C. (2008). Stratified epithelial sheets engineered from a single adult murine corneal/limbal progenitor cell. J. Cell. Mol. Med..

[16] Kosaka N., Iguchi H., Yoshioka Y., Takeshita F., Matsuki Y., Ochiya T. (2010). Secretory mechanisms and intercellular transfer of microRNAs in living cells. J. Biol. Chem..

[17] (2015). Gene Ontology Consortium. Gene Ontology Consortium: Going forward. Nucleic Acids Res..

[18] Tsubota K., Satake Y., Shimazaki J. (1996). Treatment of severe dry eye. Lancet.

[19] Mishima S. (1965). Some Physiological Aspects of the Precorneal Tear Film. Arch. Ophthalmol..

[20] Yokoi N., Uchino M., Uchino Y., Dogru M., Kawashima M., Komuro A., Sonomura Y., Kato H., Tsubota K., Kinoshita S. (2015). Importance of tear film instability in dry eye disease in office workers using visual display terminals: The Osaka study. Am. J. Ophthalmol..

[21] Uchino M., Nishiwaki Y., Michikawa T., Shirakawa K., Kuwahara E., Yamada M., Dogru M., Schaumberg D.A., Kawakita T., Takebayashi T. (2011). Prevalence and risk factors of dry eye disease in Japan: Koumi study. Ophthalmology.

[22] Ammar D.A., Noecker R.J., Kahook M.Y. (2011). Effects of benzalkonium chloride- and polyquad-preserved combination glaucoma medications on cultured human ocular surface cells. Adv. Ther..

[23] Raposo G., Nijman H.W., Stoorvogel W., Liejendekker R., Harding C.V., Melief C.J., Geuze H.J. (1996). B lymphocytes secrete antigen-presenting vesicles. J. Exp. Med..

[24] Caby M.P., Lankar D., Vincendeau-Scherrer C., Raposo G., Bonnerot C. (2005). Exosomal-like vesicles are present in human blood plasma. Int. Immunol..

[25] Pisitkun T., Shen R.F., Knepper M.A. (2004). Identification and proteomic profiling of exosomes in human urine. Proc. Natl. Acad. Sci. USA.

[26] Ogawa Y., Kanai-Azuma M., Akimoto Y., Kawakami H., Yanoshita R. (2008). Exosome-like vesicles with dipeptidyl peptidase IV in human saliva. Biol. Pharm. Bull..

[27] Kosaka N., Izumi H., Sekine K., Ochiya T. (2010). microRNA as a new immune-regulatory agent in breast milk. Silence.

[28] Street J.M., Barran P.E., Mackay C.L., Weidt S., Balmforth C., Walsh T.S., Chalmers R.T., Webb D.J., Dear J.W. (2012). Identification and proteomic profiling of exosomes in human cerebrospinal fluid. J. Transl. Med..

[29] Gui Y., Liu H., Zhang L., Lv W., Hu X. (2015). Altered microRNA profiles in cerebrospinal fluid exosome in Parkinson disease and Alzheimer disease. Oncotarget.

[30] Madison M.N., Roller R.J., Okeoma C.M. (2014). Human semen contains exosomes with potent anti-HIV-1 activity. Retrovirology.

[31] Runz S., Keller S., Rupp C., Stoeck A., Issa Y., Koensgen D., Mustea A., Sehouli J., Kristiansen G., Altevogt P. (2007). Malignant ascites-derived exosomes of ovarian carcinoma patients contain CD24 and EpCAM. Gynecol. Oncol..

[32] Inubushi S., Kawaguchi H., Mizumoto S., Kunihisa T., Baba M., Kitayama Y., Takeuchi T., Hoffman R.M., Tanino H., Sasaki R. (2020). Oncogenic miRNAs Identified in Tear Exosomes From Metastatic Breast Cancer Patients. Anticancer Res..

[33] Bjordal O., Norheim K.B., Rodahl E., Jonsson R., Omdal R. (2020). Primary Sjogren’s syndrome and the eye. Surv. Ophthalmol..

[34] Le M.T., Hamar P., Guo C., Basar E., Perdigao-Henriques R., Balaj L., Lieberman J. (2014). miR-200-containing extracellular vesicles promote breast cancer cell metastasis. J. Clin. Investig..

[35] Lakkaraju A., Rodriguez-Boulan E. (2008). Itinerant exosomes: Emerging roles in cell and tissue polarity. Trends Cell Biol..

[36] Sadier A., Viriot L., Pantalacci S., Laudet V. (2014). The ectodysplasin pathway: From diseases to adaptations. Trends Genet. TIG.

[37] Mulcahy L.A., Pink R.C., Carter D.R. (2014). Routes and mechanisms of extracellular vesicle uptake. J. Extracell. Vesicles.

[38] Svensson K.J., Christianson H.C., Wittrup A., Bourseau-Guilmain E., Lindqvist E., Svensson L.M., Morgelin M., Belting M. (2013). Exosome uptake depends on ERK1/2-heat shock protein 27 signaling and lipid Raft-mediated endocytosis negatively regulated by caveolin-1. J. Biol. Chem..

[39] Tkach M., Thery C. (2016). Communication by Extracellular Vesicles: Where We Are and Where We Need to Go. Cell.

[40] Hirayama M., Ogawa M., Oshima M., Sekine Y., Ishida K., Yamashita K., Ikeda K., Shimmura S., Kawakita T., Tsubota K. (2013). Functional lacrimal gland regeneration by transplantation of a bioengineered organ germ. Nat. Commun..

[41] Greening D.W., Xu R., Ji H., Tauro B.J., Simpson R.J. (2015). A protocol for exosome isolation and characterization: Evaluation of ultracentrifugation, density-gradient separation, and immunoaffinity capture methods. Methods Mol. Biol..

